# Supplementary material for: NLRP7 Enhances Choriocarcinoma Cell Survival and Camouflage in an Inflammasome Independent Pathway
Source: Cells. 2023 Mar 9;12(6):857. doi: 10.3390/cells12060857 (PMC10099745; doi:10.3390/cells12060857)
Supplement: Supplementary file 1 [file cells-12-00857-s001.zip › cells-2197258 supplementary.pdf]

NLRP7 Enhances Choriocarcinoma Cell Survival and Camouflage in an Inflammasome Independent Pathway

Déborah Reynaud [1,2,3,†], Nadia Alfaidy [1,2,3,*,†], Constance Collet [1,2,3], Nicolas Lemaitre [1,2,3], Frederic Sergent [1,2,3], Céline Miege [1,2,3], Emmanuelle Soleilhac [4], Alaa Al Assi [5], Padma Murthi [6,7], Gilles Courtois 4 Marie-Odile Fauvarque [4], Rima Slim [8], Mohamed Benharouga [1,2,3,‡] and Roland Abi Nahed [1,2,3,5,*,‡]

1 Institut National de la Santé et de la Recherche Médicale U1292, Biologie et Biotechnologie pour la Santé, Grenoble, France
2 Commissariat à l'Energie Atomique et aux Energies Alternatives (CEA), Biosciences and Biotechnology Institute of Grenoble 38054, France
3 Service Obstétrique, University Grenoble Alpes and Centre Hospitalo-Universitaire Grenoble Alpes, CS 10217, CEDEX 9, 38043 Grenoble, France
4 Univeristy Grenoble Alpes, Inserm, CEA, UA13 BGE, 38000 Grenoble, France
5 Laboratory of Fundamental and Applied Bioenergetics (LBFA), Univeristy Grenoble Alpes, Inserm, 38000 Grenoble, France
6 Department of Pharmacology, Monash Biomedicine Discovery Institute, Monash, Clayton, VIC 3168, Australia
7 Department of Obstetrics and Gynecology, The University of Melbourne, Parkville, VIC 3010, Australia
8 Departments of Human Genetics and Obstetrics and Gynecology, McGill University Health Centre Research Institute, Montréal, QC H4A 3J1, Canada
* Correspondence: nadia.alfaidy-benharouga@cea.fr (N.A.); rolandabinahed@gmail.com (R.A.N.); Tel.: +33-6-3207-3234 (N.A.); 33-7-702-7-1704 (R.A.N.)
† These authors contributed equally to this work.
‡ These authors contributed equally to this work.

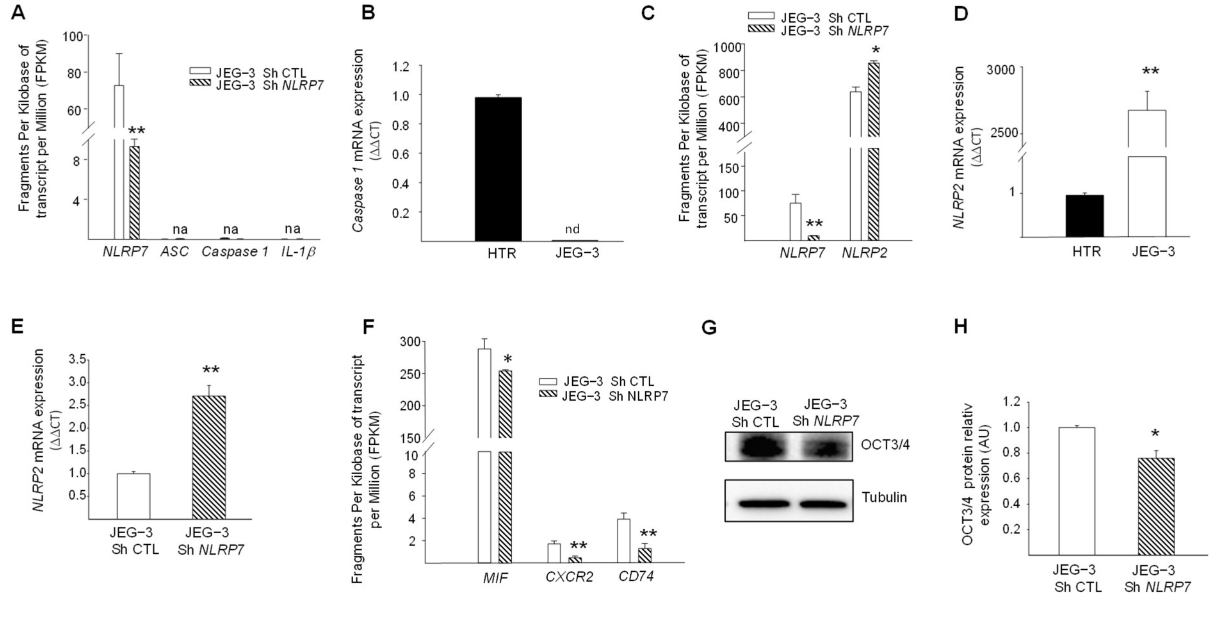

Figure S1: Validation of the differential expression of some genes selected upon RNA-sequencing in JEG-3 Sh CTL and JEG-3 Sh NLRP7. Panel A reports a comparison of Fragments per Kilobase of transcription per Million (FPKM) in gene expression levels of NLRP7, ASC, CASP 1 and IL-1β in JEG-3 Sh CTL and JEG-3 Sh NLRP7 (n = 3); ** $p < 0.01$, na: not applicable, ns: not significant. Panel B reports a comparison of the mRNA levels of CASP1 expression in HTR8/SVneo and JEG-3 cells (n = 3); nd: not detectable. Panel C comparison of Fragments per Kilobase of transcription per Million (FPKM) in gene expression levels of NLRP7 and NLRP2 (n = 3); * $p < 0.05$, ** $p < 0.01$. Panel D reports a comparison of the mRNA levels of NLRP2 expression in HTR8/SVneo and JEG-3 cells (n = 3); ** $p < 0.01$. Panel E reports a comparison of the mRNA levels of NLRP2 expression in JEG-3 Sh CTL and JEG-3 Sh NLRP7 (n = 3); ** $p < 0.01$. Panel F comparison of Fragments per Kilobase of transcription per Million (FPKM) in gene expression levels of MIF, CXCR2 and CD74 in JEG-3 Sh CTL and JEG-3 Sh NLRP7 (n = 3); * $p < 0.05$, ** $p < 0.01$. Panel G reports a comparison of OCT3/4 protein levels in JEG-3 Sh CTL and JEG-3 Sh NLRP7 cells. Panel H reports a quantification of the OCT3/4 protein levels (n = 3); * $p < 0.05$. Standardization of protein signals was performed using antibodies against tubulin.

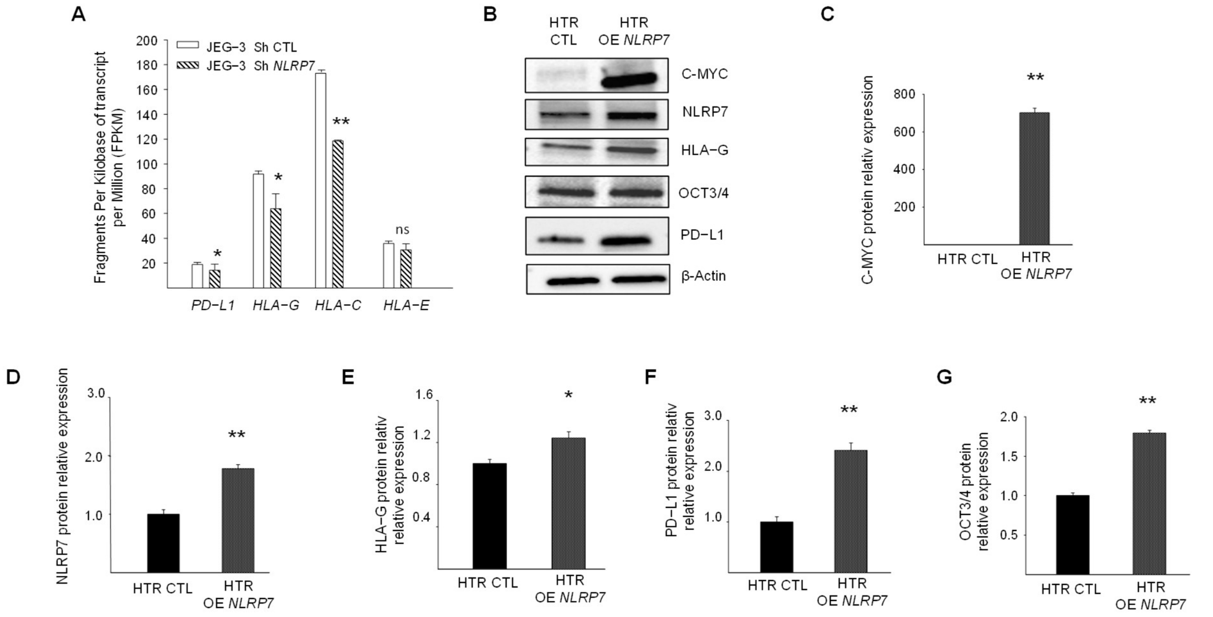

Figure S2: Characterization of NLRP7 involvement in cell camouflage. Panel A comparison of Fragments per Kilobase of transcription per Million (FPKM) in gene ex-pression levels of PD-L1, HLA-G, HLA-C and HLA-E in JEG-3 Sh CTL and JEG-3 Sh NLRP7 (n = 3); * $p < 0.05$, ** $p < 0.01$, ns: not significant. Panel B reports the comparison of protein levels in HTR CTL and HTR-OE NLRP7 cells. Membranes were incubated with antibodies to detect C-MYC, NLRP7, HLA-G, OCT3/4 and PD-L1. β-Actin was used as a loading control (n = 3). Panel C reports a quantification of the C-MYC protein levels extracted from HTR CTL and HTR-OE NLRP7 (n = 3); ** $p < 0.01$. Panel D reports a quantification of the NLRP7 protein levels extracted from HTR CTL and HTR-OE NLRP7 cells (n = 3); ** $p < 0.01$. Panel E reports a quantification of the HLA-G protein levels extracted from HTR8/SVneo CTL and HTR8/SVneo OE NLRP7 (n = 3); * $p < 0.05$. Panel F reports a quantification of the PD-L1 protein levels extracted from HTR CTL and HTR-OE NLRP7 cells (n = 3); ** $p < 0.01$. Panel G reports a quantification of the OCT3/4 protein levels extracted from HTR CTL and HTR-OE NLRP7 cells (n = 3); ** $p < 0.01$.
